# P-2222. Optimization And Performance Assessment Of A Novel Assay In The Diagnosis Of Early Lyme Disease

**DOI:** 10.1093/ofid/ofae631.2376

**Published:** 2025-01-29

**Authors:** Fabrizio Bonelli, Ashli Rode, Claudia Zierold, Jenny Howard, Nadia Allen, Giorgio Ghignoni

**Affiliations:** Diasorin SpA, Saluggia, Piemonte, Italy; Diasorin Inc., Stillwater, Minnesota; Diasorin SpA, Saluggia, Piemonte, Italy; QIAGEN, Germantown, Maryland; QIAGEN, Germantown, Maryland; Diasorin SpA, Saluggia, Piemonte, Italy

## Abstract

**Background:**

There is a need for sensitive clinical diagnostics for early and acute infection as current serology-based tests have low sensitivities in the early phases of the disease and cannot discriminate between acute and past infections. In 2016, using a 33 peptide T-cell stimulating cocktail (QIAGEN prototype), Callister et al. provided the first published evidence showing that the integration of humoral and cell mediated immunity can result in earlier detection of Lyme Disease. Additional data have since shown that interferon γ (IFNγ) release is a marker of acute disease and decreases following antibiotic treatment of the patients.

**Methods:**

A fully automated chemiluminescence immunoassay was developed that allows the detection of IgG, IgM, and an IFNγ release assay component (QFL) using a proprietary peptide-based technology; an algorithm combines the 3 results into a POS, NEG or IND call. The performance of the assay was compared to standard two-tiered testing (sTTT) in a prospective clinical trial (NCT05041595).

**Results:**

In 2021, 54 patients with sign and symptoms consistent with Lyme disease were evaluated, as well as 200 healthy subjects from an area of low Lyme incidence. The assay showed significant sensitivity improvement over sTTT (79.6% vs 57.4%). However, suboptimal specificity was observed (89.5%) with some relatively high IFNγ values that required reassessment of the test. A troubleshooting process identified two decorin binding protein peptides as being responsible for most of the non-specific reactivity of QFL. The performance of an updated assay version with a 31-peptide QFL stimulation tube was evaluated in 2022 on 56 patients with *erythema migrans,* and 99 apparently healthy subjects from endemic areas. Sensitivity and specificity were assessed and compared to sTTT and found to be 73.2% vs 58.9%, and 100% for both, respectively.

**Conclusion:**

The data presented support the operational utility of the assay as an aid in the diagnosis of early Lyme disease, while providing high specificity after the optimization of the stimulation peptides of the QFL assay component. A promising expanded clinical validation study for the assay is in process for submission to the U.S. Food and Drug Administration.

**Disclosures:**

Fabrizio Bonelli, PhD, Diasorin SpA: employee Ashli Rode, MS, Diasorin Inc: employee Claudia Zierold, PhD, Diasorin SpA: Advisor/Consultant Jenny Howard, PhD, QIAGEN: Employee|QIAGEN: Stocks/Bonds (Public Company) Nadia Allen, PhD, QIAGEN: employee|QIAGEN: Stocks/Bonds (Public Company) Giorgio Ghignoni, MD, Diasorin: Employee

